# Health consciousness and pro-environmental behaviors in an Italian representative sample: A cross-sectional study

**DOI:** 10.1038/s41598-023-35969-w

**Published:** 2023-05-31

**Authors:** Greta Castellini, Marta Acampora, Livio Provenzi, Lucia Cagliero, Luigi Lucini, Serena Barello

**Affiliations:** 1 EngageMinds HUB – Consumer, Food & Health Engagement Research Center, Università Cattolica del Sacro Cuore, Milan, Italy; 2 Faculty of Agriculture, Food and Environmental Sciences, Università Cattolica del Sacro Cuore, Via Bissolati, 74, 26100 Cremona, Italy; 3 Department of Psychology, Università Cattolica del Sacro Cuore, L.go Gemelli 1, 20123 Milan, Italy; 4 Department of Brain and Behavioral Sciences, University of Pavia, Pavia, Italy; 5 Developmental Psychobiology Lab, IRCCS Mondino Foundation, Pavia, Italy; 6 Department for Sustainable Food Process, Università Cattolica del Sacro Cuore, 29122 Piacenza, Italy; 7 Developmental Psychobiology Lab, Department of Brain and Behavioral Sciences, University of Pavia, via Mondino 2, 27100 Pavia, PV Italy

**Keywords:** Climate-change mitigation, Public health

## Abstract

Individual health-related behavior is among the most influential yet modifiable factors affecting both climate change and chronic disease. To encourage behaviors bringing about environmental and health co-benefits, it is important to understand the underlying factors of behavior change for healthy and sustainable lifestyles. One area of potential overlap concerns people’s health consciousness. The purpose of this study was to examine the relationship between health consciousness and pro-environmental behavior. We investigated whether health consciousness correlates with five clusters of pro-environmental behaviors: sustainable food consumption, recycling, green purchasing, sustainable mobility, and energy saving. Research data were collected via cross-sectional survey involving a representative sample of n = 1011 Italian citizens. Statistically significant differences emerged in the frequency of the different classes of pro-environmental behaviors: people living in Italy most frequently implement sustainable behaviors related to energy saving and recycling while sustainable mobility behaviors are the least implemented. Moreover, the stepwise linear regression model demonstrated the predictive role of citizens’ health consciousness on the adoption of specific classes of pro-environmental behaviors showing how higher involvement in one’s own health determines higher levels of pro-environmental behaviors. These results highlight the relevance of developing and testing complex programs featuring educational, sensitization, and structural strategies to increase citizens involvement in public health and pro-environmental behaviors.

## Introduction

Characteristics of contemporary society, including over and inequitable consumption, have contributed to the increase in non-communicable diseases (NCDs). These social factors, along with continuing growth in the global population, led to heightened atmospheric concentrations of greenhouse gases (GHGs) and to climate change with a substantial increase in severe heatwaves, droughts, storms, and floods^1^. These environmental changes will bring greater risk to human survival and will likely worsen the incidence of some NCDs, including cardiovascular disease, some cancers, respiratory health, mental disorders, injuries, and malnutrition. The dual crises of mitigating the effect of climate change and controlling NCDs are therefore recognized as two of the greatest and most urgent contemporary global health challenges and they would benefit from the alignment of their policy agendas, offering synergistic opportunities to improve population and planetary health^2^.

It is increasingly understood that mitigating climate change and reducing the incidence of chronic diseases require both high-level policy actions as well as individual behavior changes. Indeed, the practical adoption of pro-environmental behaviors is shaped by a complex intertwining of multiple determinants such as demographic factors, external factors (e.g. institutional, economic, social, and cultural), and individual attitudes and motivations (e.g. knowledge, awareness, values, emotions, locus of control, and priorities)^3–5^. In particular, individual behavior is one of the most influential factors which affect both environmental change and chronic disease development^6–8^. As a result, for the benefit of the environment, it is critical to build long-term policies and interventions that give greater possibilities for citizens to engage in healthy and pro-environmental behaviors. In particular, the current historical moment offers a favorable context for this type of intervention. In fact, as demonstrated by previous studies^7,8^ the COVID-19 emergency has fostered an increasing public awareness of the link between environmental issues and human health, supporting the “One Health” perspective that identifies the health of people as closely connected to the health of animals and the environmental themes^9^.

The pressing urgency to tackle both environmental and health challenges, supported by the historical moment in which citizens are aware of this connection, reinforces the urgent need for globally operationalizing a “planetary health approach” and connecting environmental and health sciences^10^. By emphasizing interconnections between human health and environmental changes and enabling holistic thinking about overlapping challenges and integrated solutions for present and future generations, the concept of planetary health offers an opportunity to advance the 2030 Agenda for Sustainable Development^11^. Including the identification of co-benefits across targets, encouraging effective cross-sector action and partnerships, and ensuring policy coherence. The adoption of the 2030 Agenda for Sustainable Development in 2015 created new chances to work toward healthier environments by utilizing ‘whole-of-government’ and ‘whole-of-society’ approaches. It established a solid policy framework that recognizes health as a result and serves as a facilitator of sustainable policies across all levels of government. In this scenario, citizens assume an unprecedented role in placing health and equity at the heart of the political agenda. This demands new governance models that focus on the people active role in contributing to make human and environmental health more sustainable. It also requires enhancing capacities among health professionals to embrace this new level of complexity, understand the multiple links between sectoral policies and health, and successfully engage with other government sectors and stakeholders^12^. However, despite the links between the environment and health are recognized, policy actions designed to promote sustainable behavior are rarely framed in terms of human health and strategies to change health behavior are not often applied to environmental behavior.

### The present study

Increasing research evidence suggests the possibility of environmental and health co-benefits, by facilitating the adoption of sustainable specific behaviors. Thus, to encourage behaviors bringing about environmental and health co-benefits, it is important to understand underlying factors that create behavior change for healthy and sustainable lifestyles. One area of potential overlap concerns people's health consciousness. Health consciousness refers to a psychological state where an individual is aware of and involved in his/her health condition. However, to date, this variable has been poorly investigated in relation to pro-environmental behaviors^13,14^, and no study has been conducted in the Italian context. Thus, the purpose of the present study was to explore the relationship between health consciousness and a set of pro-environmental behaviors. Specifically, we investigated whether health consciousness is correlated with five clusters of specific pro-environmental behaviors: sustainable food consumption, recycling, green purchasing, sustainable mobility, and energy saving.

## Material and methods

### Sample and procedure

Research data were collected via a questionnaire that was filled out by a representative sample of the Italian population with sex, age, profession, level of education, size of the center, and geographical area extracted by stratified sampling. The percentages relating to the Italian population were taken from the website of ISTAT (https://www.istat.it/it/). The survey was conducted using a CAWI (Computer Assisted Web Interviewing) methodology between 09 May 2022 and 13 May 2022. The sample consists of 1011 participants randomly selected from the consumers’ panel managed by Norstat srl (https://norstat.it/). In order to check the reliability of the sample, control questions were included within the questionnaire. Specifically, the participants were asked to answer some questions, measured on Likert scales, with the option "neither agree nor disagree". Data analysis was carried out considering only those people who had passed these control questions. This study is part of the broader CLIMAL project (D.3.2 2021 funding program, Università Cattolica del Sacro Cuore) aimed at assessing the psychosocial impact of climate change and promoting citizen engagement towards sustainable behaviors. This study has been performed in accordance with the Declaration of Helsinki and has been approved by an independent ethics committee of Università Cattolica del Sacro Cuore in Milan (CERPS). Informed consent was obtained from all subjects.

### Questionnaire

Socio-demographic questions such as gender, age, place of residence, work, and level of education. Twenty-one questions about the frequency with which sustainable behavior is carried out in everyday life. The behaviors concern: food (beh_food), energy (beh_ener), green purchasing (beh_sust), recycling (beh_recy) and mobility (beh_mobi). Answers were assessed on a 7-step Likert-type scale (1 = never; 7 = always), where higher numbers corresponded to higher frequency. Question items are, for example: “*I try to reduce the consumption of foods from animal origin”; “I turn off lights when I leave a room; I buy brands which I know are sustainable”; “I carefully recycle; I move around on foot, by bicycle or public transport rather than using private vehicles such as cars and scooters*”. Health Consciousness Scale^15^. Eleven questions concerning attitudes towards one's personal health. Answers were assessed on a 7-step Likert-type scale, where higher numbers corresponded to higher levels of agreement. Question items are, for example: *“I’m concerned about my health all the time; Living life without disease and illness is very important to me”.* The questionnaire provides three subscales (range: 1–7): self-health awareness (hc_shawa), personal responsibility (hc_presp), and health motivation (hc_hmoti). An health consciousness total score (range: 1–7) is also obtained (hc_score).

### Plan of analysis

Repeated-measure analysis of variance was conducted to assess the presence of statistically significant differences in the frequency of the different classes of pro-environmental behaviors, controlling by gender and age. Preliminary Pearson’s bivariate correlations were used to explore the presence of associations among the different classes of pro-environmental behaviors (i.e. beh_food, beh_ener, beh_sust, beh_recy, beh_mobi). Similarly, Pearson’s bivariate correlations were computed to assess the association of the health consciousness total score (i.e. hc_score) and subscales (i.e. hc_shawa, hc_presp, hc_hmoti) with each class of pro-environmental behaviors (i.e. beh_food, beh_ener, beh_sust, beh_recy, beh_mobi). To test the predictive role of citizens’ health consciousness total score on the adoption of specific different classes of pro-environmental behaviors a stepwise linear regression model were built as follows: step 1, socio-demographic variables (i.e. gender, age); step 2, simple hc_score effect; step 3, interactive gender*hc_score and age*hc_score effects. For each step, the model fit was estimated using *R*^2^ coefficients and the relative step-by-step increase in model fit was estimated using Δ*R*^2^. Standardized coefficients (*Beta*) were computed for each sample and interactive effect, with the relative 95% confidence intervals. The statistical analyses were performed with Jamovi 2.2.5 for Windows 11. In light of the large sample size, a conservative approach was adopted by considering significant only correlations at *p* < 0.01 and moderate-to-high *r* coefficients > 0.35 and mean comparisons at *p* < 0.01 and effect size η^2^_p_ > 0.10.

### Ethics

Informed consent was obtained from all subjects.

## Results

### Sample description

1011 adults aged between 20 and 72 years old were involved. Gender was equally distributed in the final sample (49.8% males, 50.2% females). Age was stratified based on the actual Italian population distribution as follows: 265 subjects below 35 years (26.2%), 440 subjects between 35 and 54 years (43.5%), and 306 subjects above 54 years (30.3%). Participants were also distributed by geographical areas to have a representative sample of respondents: 256 subjects from North-West regions (25.3%), 189 from North-East (18.7%), 206 from Center regions (20.4%), 360 from South and Island regions (35.6%). The educational level was also stratified according to the actual Italian population distribution as follows: no educational title, n = 8 (0.8%), elementary school, n = 11 (1.1%), lower secondary school, n = 170 (16.8%), higher secondary school, n = 575 (56.9%), academic diploma or higher, n = 247 (24.4%). Finally, 16.8% of respondents were unemployed. Descriptive statistics for pro-environmental behaviors and health consciousness subscale and total score are reported in Table 1.

**Table 1:** Descriptive statistics.

	Mean	SD	Min	Max	Skewness		Kurtosis	
					Value	SE	Value	SE
Pro-environmental behaviors related to								
Food (beh_food)	5.16	1.01	1.00	7.00	− 0.57	0.08	0.75	0.15
Energy (beh_ener)	5.82	1.07	1.00	7.00	− 1.09	0.08	1.19	0.15
Green purchasing (beh_sust)	4.57	1.00	1.00	7.00	− 0.19	0.08	0.43	0.15
Recycling (beh_recy)	5.60	1.08	1.00	7.00	− 0.86	0.08	0.96	0.15
Mobility (beh_moby)	3.83	1.24	1.00	7.00	0.02	0.08	0.01	0.15
Health consciousness scores								
Total (hc_score)	5.22	0.95	1.50	7.00	− 0.26	0.08	− 0.13	0.15
Self-health awareness (hc_shawa)	5.07	1.15	1.00	7.00	− 0.30	0.08	− 0.08	0.15
Personal responsability (hc_presp)	5.00	1.03	1.75	7.00	− 0.07	0.08	0.01	0.15
Health motivation (hc_hmoti)	5.59	1.11	1.00	7.00	− 0.62	0.08	0.10	0.15

### Main findings

**Figure 1:**
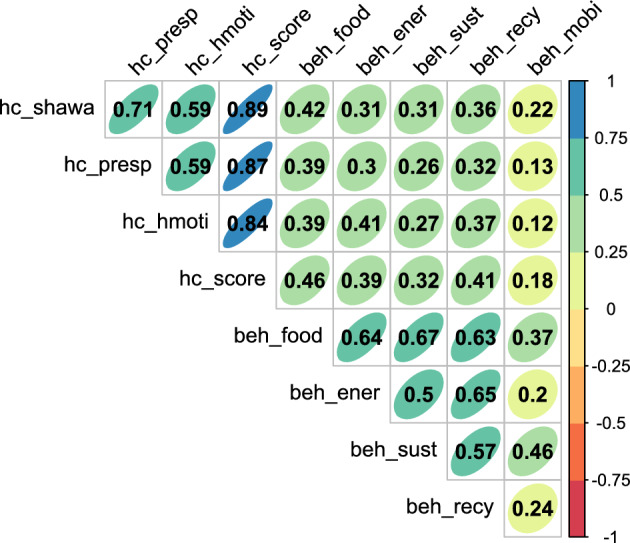
Correlation matrix for pro-environmental behaviors and health consciousness subscale and total scores. Pro-environmental behaviors related to food (beh_food), energy (beh_ener), green purchasing (beh_sust), recycling (beh_recy), mobility (beh_moby). Health consciousness total score (hc_score), self-health awareness (hc_shawa), personal responsibility (hc_presp), health motivation (hc_hmoti).

Statistically significant differences emerged in the frequency of the different classes of pro-environmental behaviors, *F*(4, 4028) = 1047.48, *p* < 0.001, η^2^_p_ = 0.51: beh_ener and beh_recy had the highest frequency, followed by beh_food, beh_sust, and beh_mobi. Although significant effects emerged for gender and age (Supplementary Table S1), these effects were below the effect size threshold of η^2^_p_ > 0.10 and they are not further discussed here. The correlations of the different classes of pro-environmental behaviors with health consciousness total score and subscales are reported in a heath map in Fig. 1.

Significant and moderate-to-robust correlations emerged for beh_food with hc_shawa, *r* = 0.42, *p* < 0.001, hc_presp, *r* = 0.39, *p* < 0.001, hc_hmoti, *r* = 0.39, *p* < 0.001, and hc_score, *r* = 0.46, *p* < 0.001; for beh_ener with hc_hmoti, *r* = 0.41, *p* < 0.001 and hc_score, *r* = 0.39, *p* < 0.001; for beh_recy with hc_shawa, *r* = 0.36, *p* < 0.001, hc_hmoti, *r* = 0.37, *p* < 0.001, and hc_score, *r* = 0.41, *p* < 0.001. No significant correlations emerged with health consciousness total score and subscales for beh_sust and beh_mobi.

**Figure 2:**
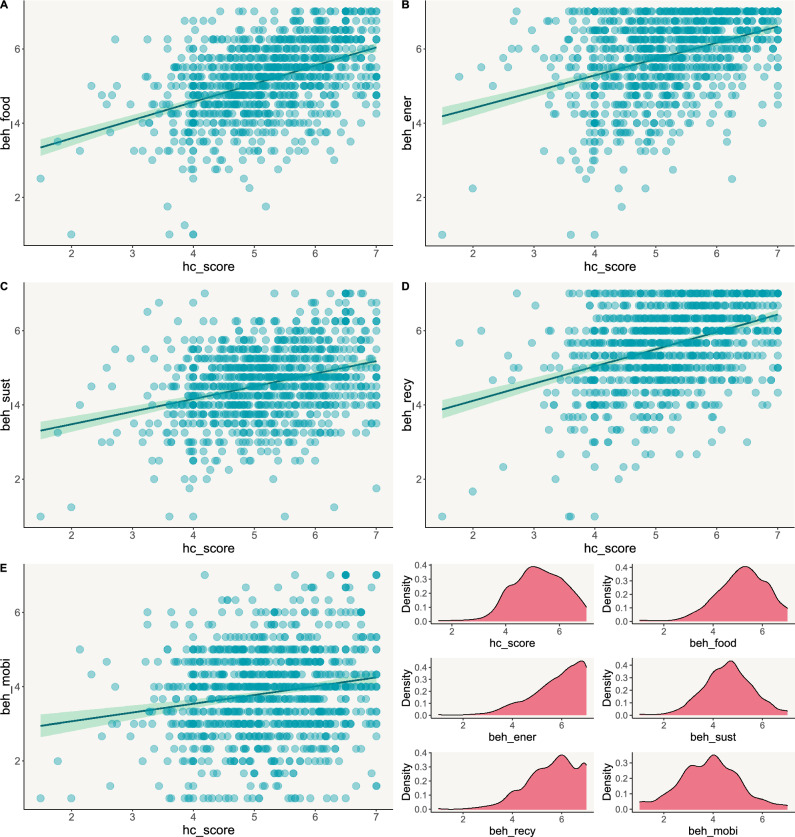
The association between health consciousness (hc_score) and different pro-environmental behaviors related to (**A**) food, (**B**) energy, (**C**) green purchasing, (**D**) recycling, (**E**) mobility. Pro-environmental behaviors related to food (beh_food), energy (beh_ener), green purchasing (beh_sust), recycling (beh_recy), mobility (beh_moby). Health consciousness total score (hc_score). The right bottom panel reports the density graphs depicting variables distribution.

**Figure 3:**
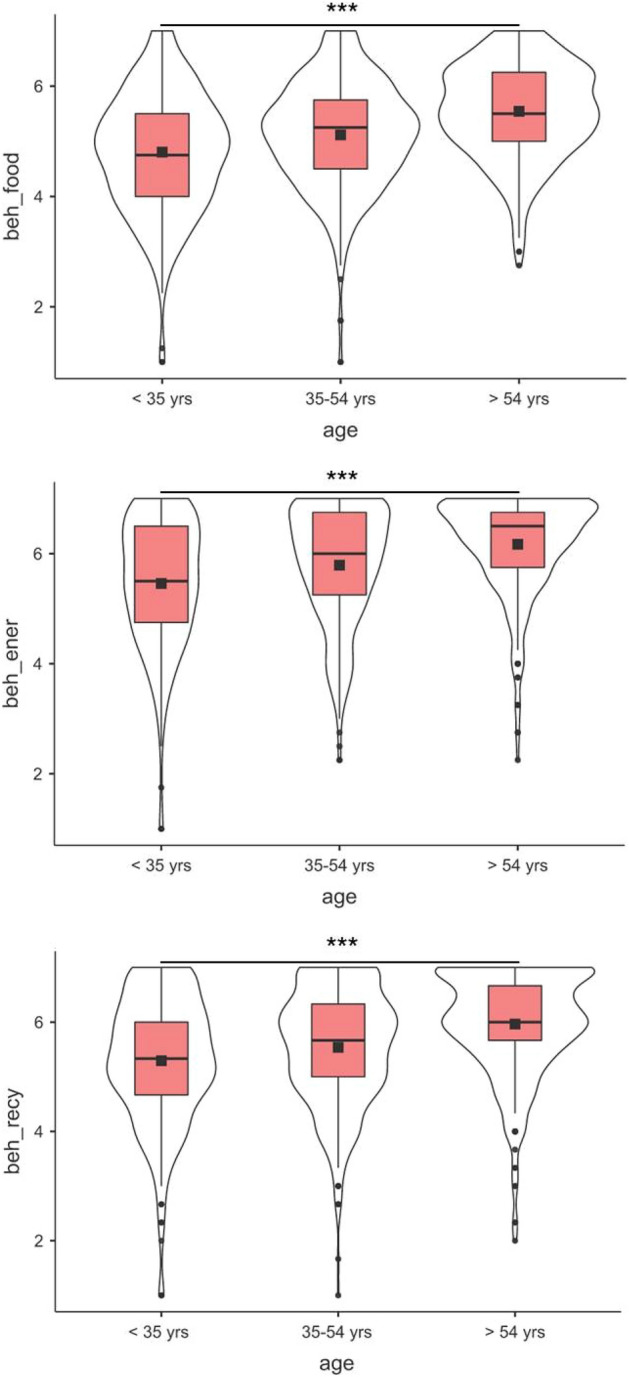
Pro-environmental behaviors by age of respondents. ***p < 0.001.

The hierarchical steps of the regression analyses are reported in the Supplementary Table S2. Table 2 reports the final regression models including all independent and joint effects for the five separate classes of pro-environmental behaviors. The final model explained 27% of variance for beh_food, *F*(7, 1003) = 55.49, *p* < 0.001, 20% of variance for beh_ener, *F*(7, 1003) = 37.04, *p* < 0.001, 11% of variance for beh_sust, *F*(7, 1003) = 19.73, *p* < 0.001, 20% of variance for beh_recy, *F*(7, 1003) = 38.04, *p* < 0.001, and 3% of variance for beh_mobi, *F*(7, 1003) = 5.22, *p* < 0.001. For all the target classes of pro-environmental behaviors, hc_score was a significant, positive, and independent predictor (Fig. 2). Additionally, age was significantly associated with beh_food, beh_ener, and beh_recy (Fig. 3): for these classes of pro-environmental behaviors, older respondents (age > 54 years) had higher scores compared to younger counterparts (age < 35 years). No significant interactive effects (*p* < 0.01) emerged.

**Table 2:** Regression models.

Predictors	*t*	*p*	*Beta*	95% confidence interval	
				Lower	Upper
(A) beh_food					
Intercept	6.780	< 0.001			
Gender (female vs. male)	1.153	0.249	0.212	0.106	0.318
Age (35–54 yrs vs. < 35 yrs)	1.334	0.183	0.240	0.109	0.371
Age (> 54 yrs vs. < 35 yrs)	2.629	0.009	0.607	0.465	0.749
hc_score	8.473	< 0.001	0.480	0.369	0.591
Age (35–54 yrs vs. < 35 yrs) * hc_score	− 0.674	0.501	− 0.044	− 0.173	0.085
Age (> 54 yrs vs. < 35 yrs) * hc_score	− 1.102	0.271	− 0.078	− 0.216	0.061
Gender (female vs. male) * hc_score	− 0.461	0.645	− 0.025	− 0.131	0.081
**(**B) beh_ener					
Intercept	8.273	< 0.001			
Gender (female vs. male)	0.567	0.571	0.096	− 0.015	0.208
Age (35–54 yrs vs. < 35 yrs)	1.783	0.075	0.249	0.111	0.386
Age (> 54 yrs vs. < 35 yrs)	3.401	< 0.001	0.568	0.419	0.716
hc_score	7.583	< 0.001	0.451	0.334	0.567
Age (35–54 yrs vs. < 35 yrs) * hc_score	− 1.133	0.258	− 0.078	− 0.214	0.057
Age (> 54 yrs vs. < 35 yrs) * hc_score	− 2.058	0.040	− 0.152	− 0.297	− 0.007
Gender (female vs. male) * hc_score	− 0.269	0.788	− 0.015	− 0.127	0.096
(C) beh_sust					
Intercept	7.6291	< 0.001			
Gender (female vs. male)	0.9421	0.346	0.050	− 0.067	0.167
Age (35–54 yrs vs. < 35 yrs)	− 0.0377	0.970	0.139	− 0.006	0.283
Age (> 54 yrs vs. < 35 yrs)	1.1276	0.260	0.320	0.164	0.477
hc_score	5.2899	< 0.001	0.331	0.208	0.453
Age (35–54 yrs vs. < 35 yrs) * hc_score	0.3848	0.700	0.028	− 0.114	0.170
Age (> 54 yrs vs. < 35 yrs) * hc_score	− 0.396	0.692	− 0.031	− 0.183	0.122
Gender (female vs. male) * hc_score	− 0.8072	0.420	− 0.048	− 0.166	0.069
(D) beh_recy					
Intercept	7.682	< 0.001			
Gender (female vs. male)	− 0.163	0.870	0.019	− 0.093	0.130
Age (35–54 yrs vs. < 35 yrs)	1.147	0.252	0.162	0.025	0.299
Age (> 54 yrs vs. < 35 yrs)	3.441	< 0.001	0.523	0.374	0.671
hc_score	7.591	< 0.001	0.450	0.334	0.566
Age (35–54 yrs vs. < 35 yrs) * hc_score	− 0.721	0.471	− 0.050	− 0.185	0.085
Age (> 54 yrs vs. < 35 yrs) * hc_score	− 2.205	0.028	− 0.163	− 0.307	− 0.018
Gender (female vs. male) * hc_score	0.225	0.822	0.013	− 0.099	0.124
(E) beh_mobi					
Intercept	5.126	< 0 .001			
Gender (female vs. male)	0.655	0.512	− 0.056	− 0.179	0.067
Age (35–54 yrs vs. < 35 yrs)	0.345	0.730	− 0.008	− 0.159	0.143
Age (> 54 yrs vs. < 35 yrs)	0.665	0.506	− 0.035	− 0.199	0.129
hc_score	3.692	< 0 .001	0.242	0.113	0.370
Age (35–54 yrs vs. < 35 yrs) * hc_score	− 0.365	0.715	− 0.028	− 0.177	0.121
Age (> 54 yrs vs. < 35 yrs) * hc_score	− 0.754	0.451	− 0.061	− 0.221	0.098
Gender (female vs. male) * hc_score	− 0.828	0.408	− 0.052	− 0.175	0.071

Pro-environmental behaviors related to food (beh_food), energy (beh_ener), green purchasing (beh_sust), recycling (beh_recy), mobility (beh_moby). Health consciousness total score (hc_score).

## Discussion

This study aimed to explore the link between health-related attitudes and pro-environmental behaviors in a large sample representative of Italian population.

The results showed that people living in Italy most frequently implement sustainable behaviors related to energy saving and recycling whereas sustainable mobility behaviors are the least implemented. These results are supported by past studies which confirmed that people living in Italy are used to enact behaviors related to energy saving^16^, by limiting the use of air conditioning, turning off lights, using the stairs instead of the elevator, and related to reusing and recycling objects by postponing their end of life^17^. However, sustainable mobility behaviors are much less frequent mainly due to external barriers that prevent their implementation such as limited services offered by the cities^18^. Indeed, research findings suggests that external barriers such as vehicle performance and range, the total cost of ownership, shortage of charging infrastructure, lack of consumer awareness about sustainable mobility technology are critically influential in sustainable mobility behaviours adoption^19,20^.

Furthermore, if we consider the link between the involvement in one's own health and the implementation of sustainable actions, we note that behaviors related to mobility were the least correlated with health consciousness. This weak link can be at least partially explained by the fact that these behaviors might not be perceived by citizens as strictly linked to the protection of the planet. Indeed, the main reasons that impact on the implementation of sustainable mobility behaviors are weakly linked to environmental concern, rather they appear to be better explained by affordability and comfort^21^. Accordingly, a previous study carried out in Italy has highlighted no significant associations between green self-identity and sustainable mobility behaviors^22^ and a further study, carried out on 28 European countries, has shown that 60% of people living in Italy choose the means of transport considering its comfort and not the environmental issue^23^. In addition, the low diffusion of sharing services in Italy^24^ and the presence of few stations where one can pick up and drop off the shared vehicle, lead the citizen to increase efforts for the realization of that behavior that negatively impact the repetition of it.

Furthermore, we noted that for all the target classes of pro-environmental behaviors, the engagement in own health was a significant, positive, and independent predictor. This finding shows that there might be a parallelism between health and pro-environment behaviors. Interesting research has linked these two issues by applying HBM (Health Belief Model) on water consumption^25^. This study showed how health risk factors positively impacted the appropriate use of water. Lindsay and Strathman^26^ also used the HBM to investigate recycling behavior reporting significant influence from several HBM factors including self-efficacy and perceived seriousness and benefits. These results highlight how it is therefore necessary to promote the involvement of people in their own health to increase the frequency of pro-environmental behaviors.

Additionally, age was significantly associated with sustainable food consumption, recycling, and energy saving. Indeed, older respondents (age > 54 years) had higher scores compared to younger counterparts (age < 35 years). This finding seems to contrast with the studies carried out so far which pointed out that young people are the most environmentally aware and therefore most determined to enact pro-environmental behaviors^27,28^. However, it is also possible to find studies that claim exactly the opposite, reporting that increase in environmentally friendly behavior and awareness is positively associated with increase in age. Linking Pro-environmental behaviors and age, it was inferred that a more mature demographic (age group 36–50) has higher degrees in displaying environmental orientation; compared to the younger adults (age group 20–35 and less)^29,30^. Similar results were found in consumption of green electricity for the mature demographic compared to younger adults^31^. From these divergent results it is possible to conclude that it is not clear how age may impact this type of behavior. What we can hypothesize is that probably the socio-demographic profile of the different participants involved in the studies plays a significant role in explaining this variance in results^32^.

Limitations of this study should be highlighted. Indeed, the frequency of sustainable behaviors was collected on self-reported items that do not allow to testifying the actual behavior. Moreover, these items were created ad hoc and therefore it is not certain that they measure what they intend to measure. This is a cross-sectional study and results cannot be interpreted in terms of causal relationships. Finally, it should be acknowledged that individual pro-environmental choices emerged from a multi-layered interactions of different determinants which only partially rely on individuals. Indeed, contextual and external barriers related to the institutional, economic, social, and cultural scenario should not be underestimated and need to be specifically addressed and put in dialogue with individual facilitators or barriers – such as health-related attitudes explored in the present study. Further research should verify the causal assumption presented in this study with a structural equation model to confirm the measurement and structural model and consider to monitor real world behaviors in order to better understand the role of people health motivations in orienting pro-environmental actions.

## Conclusions

This study suggested the presence of a significant relationship between people engagement in one's own health and specific sustainable behaviors concerning different fields (e.g. sustainable food consumption, recycling, green purchasing, sustainable mobility, and energy saving). As such, it seems plausible that promoting citizens’ involvement in their health might also favor a positive attitude toward pro-environmental behaviors. Consistently, it seems plausible to remark the opportunity of developing and testing integrated programs of educational, sensitization, and structural interventions to increase health and environmental awareness in the Italian population.

## Supplementary Information

Supplementary Table S1.

Supplementary Table S2.

## Data Availability

The datasets used and/or analyzed during the current study are available from the corresponding author on reasonable request.
