# Supplementary material for: Health consciousness and pro-environmental behaviors in an Italian representative sample: A cross-sectional study
Source: Sci Rep. 2023 May 31;13:8846. doi: 10.1038/s41598-023-35969-w (PMC10231290; doi:10.1038/s41598-023-35969-w)
Supplement: Supplementary file 1 — Supplementary Table S1. [file 41598_2023_35969_MOESM1_ESM.docx]

**Health consciousness and pro-environmental behaviors in an Italian representative sample: a cross-sectional study**

Castellini et al (2023) Scientific Reports

**Supplementary Table S1.** Detailed information on the with and between subjects effects, including post-hoc comparisons.

| Within Subjects Effects | | | | | | | | | | | | | | | | | | | | | | | | | | | | | | | | | | | | | | | | | |
| --- | --- | --- | --- | --- | --- | --- | --- | --- | --- | --- | --- | --- | --- | --- | --- | --- | --- | --- | --- | --- | --- | --- | --- | --- | --- | --- | --- | --- | --- | --- | --- | --- | --- | --- | --- | --- | --- | --- | --- | --- | --- |
|  | | | | | |  | |  | | | |  | |  | |  | | |  | | | |  | | |  | | | |  | |  | | |  | | |  | |  | |
|  | | | | | | | | **Sum of Squares** | | | | | | **df** | | | | | **Mean Square** | | | | | | | **F** | | | | | | **p** | | | | | | **η²_p_** | | | |
| beh | | | | | |  | | 2508.49 | | | |  | | 4 | |  | | | 627.12 | | | |  | | | 1047.48 | | | |  | | < .001 | | |  | | | 0.51 | |  | |
| beh ✻ gender | | | | | |  | | 14.74 | | | |  | | 4 | |  | | | 3.69 | | | |  | | | 6.16 | | | |  | | < .001 | | |  | | | 0.01 | |  | |
| beh ✻ age | | | | | |  | | 53.60 | | | |  | | 8 | |  | | | 6.70 | | | |  | | | 11.19 | | | |  | | < .001 | | |  | | | 0.02 | |  | |
| Residual | | | | | |  | | 2411.55 | | | |  | | 4028 | |  | | | 0.60 | | | |  | | |  | | | |  | |  | | |  | | |  | |  | |
| Note. Type 3 Sums of Squares | | | | | | | | | | | | | | | | | | | | | | | | | | | | | | | | | | | | | | | | | |
|  | | | | | | | | | | | | | | | | | | | | | | | | | | | | | | | | | | | | | | | | | |
| Between Subjects Effects | | | | | | | | | | | | | | | | | | | | | | | | | | | | | | | | | | | | | | | | | |
|  | |  | |  | | | | | | |  | |  | |  | |  | | | | |  | | |  | | |  | | |  | | |  | | |  | |  | | |
|  | | | | **Sum of Squares** | | | | | | | | | **df** | | | | **Mean Square** | | | | | | | | **F** | | | | | | **p** | | | | | | **η²_p_** | | | | |
| gender | |  | | 28.63 | | | | | | |  | | 1 | |  | | 28.63 | | | | |  | | | 8.94 | | |  | | | 0.003 | | |  | | | 0.01 | |  | | |
| age | |  | | 179.71 | | | | | | |  | | 2 | |  | | 89.86 | | | | |  | | | 28.04 | | |  | | | < .001 | | |  | | | 0.05 | |  | | |
| Residual | |  | | 3226.94 | | | | | | |  | | 1007 | |  | | 3.20 | | | | |  | | |  | | |  | | |  | | |  | | |  | |  | | |
| Note. Type 3 Sums of Squares | | | | | | | | | | | | | | | | | | | | | | | | | | | | | | | | | | | | | | | | | |
|  | | | | | | | | | | | | | | | | | | | | | | | | | | | | | | | | | | | | | | | | | |
| Post Hoc Comparisons - beh | | | | | | | | | | | | | | | | | | | | | | | | | | | | | | | | | | | | | | | | | |
| **Comparison** | | | | | | | | | |  | | | | | | | | | | | | | | | | | | | | | | | | | | | | | | | |
| **beh** | | |  | | | | **beh** | | | **Mean Difference** | | | | | | | | | | **SE** | | | | **df** | | | | | **t** | | | | | | | **p_bonferroni_** | | | | | |
| food |  | | - | |  | | energy | |  | -0.65 | | | | | | | |  | | 0.03 |  | | | 1007.00 | | |  | | -22.88 | | | |  | | | < .001 | | | | |  |
|  |  | | - | |  | | sust | |  | 0.59 | | | | | | | |  | | 0.03 |  | | | 1007.00 | | |  | | 22.76 | | | |  | | | < .001 | | | | |  |
|  |  | | - | |  | | recy | |  | -0.45 | | | | | | | |  | | 0.03 |  | | | 1007.00 | | |  | | -15.40 | | | |  | | | < .001 | | | | |  |
|  |  | | - | |  | | mobi | |  | 1.33 | | | | | | | |  | | 0.04 |  | | | 1007.00 | | |  | | 33.50 | | | |  | | | < .001 | | | | |  |
| energy |  | | - | |  | | sust | |  | 1.24 | | | | | | | |  | | 0.03 |  | | | 1007.00 | | |  | | 37.42 | | | |  | | | < .001 | | | | |  |
|  |  | | - | |  | | recy | |  | 0.21 | | | | | | | |  | | 0.03 |  | | | 1007.00 | | |  | | 7.15 | | | |  | | | < .001 | | | | |  |
|  |  | | - | |  | | mobi | |  | 1.98 | | | | | | | |  | | 0.05 |  | | | 1007.00 | | |  | | 42.82 | | | |  | | | < .001 | | | | |  |
| sust |  | | - | |  | | recy | |  | -1.03 | | | | | | | |  | | 0.03 |  | | | 1007.00 | | |  | | -33.38 | | | |  | | | < .001 | | | | |  |
|  |  | | - | |  | | mobi | |  | 0.74 | | | | | | | |  | | 0.04 |  | | | 1007.00 | | |  | | 19.66 | | | |  | | | < .001 | | | | |  |
| recy |  | | - | |  | | mobi | |  | 1.77 | | | | | | | |  | | 0.05 |  | | | 1007.00 | | |  | | 39.02 | | | |  | | | < .001 | | | | |  |
|  | | | | | | | | | | | | | | | | | | | | | | | | | | | | | | | | | | | | | | | | | |
