# Supplementary material for: Health consciousness and pro-environmental behaviors in an Italian representative sample: A cross-sectional study
Source: Sci Rep. 2023 May 31;13:8846. doi: 10.1038/s41598-023-35969-w (PMC10231290; doi:10.1038/s41598-023-35969-w)
Supplement: Supplementary file 2 — Supplementary Table S2. [file 41598_2023_35969_MOESM2_ESM.docx]

**Health consciousness and pro-environmental behaviors in an Italian representative sample: a cross-sectional study**

Castellini et al (2023) Scientific Reports

**Supplementary Table S2.** Detailed information the regression models.

[a] beh_food

| Model Fit Measures | | | | | | | | | | | | | | | | | | | | | | | | | | | | | | | | | |  |  |  |  |  |  |  |
| --- | --- | --- | --- | --- | --- | --- | --- | --- | --- | --- | --- | --- | --- | --- | --- | --- | --- | --- | --- | --- | --- | --- | --- | --- | --- | --- | --- | --- | --- | --- | --- | --- | --- | --- | --- | --- | --- | --- | --- | --- |
|  | | | | | | | | | | **Overall Model Test** | | | | | | | | | | | | | | | | | | | | | | | |  |  |  |  |  |  |  |
| **Model** | | **Adjusted R²** | | | | | | | | **F** | | | | | | **df1** | | | | | **df2** | | | | | | | | | **p** | | | |  |  |  |  |  |  |  |
| 1 |  | 0.10 | | | | |  | | | 37.81 | | | | |  | 3 | | |  | | 1007 | | | | |  | | | | < .001 | |  | |  |  |  |  |  |  |  |
| 2 |  | 0.28 | | | | |  | | | 96.89 | | | | |  | 4 | | |  | | 1006 | | | | |  | | | | < .001 | |  | |  |  |  |  |  |  |  |
| 3 |  | 0.27 | | | | |  | | | 55.49 | | | | |  | 7 | | |  | | 1003 | | | | |  | | | | < .001 | |  | |  |  |  |  |  |  |  |
|  | | | | | | | | | | | | | | | | | | | | | | | | | | | | | | | | | |  |  |  |  |  |  |  |
| Model Comparisons | | | | | | | | | | | | | | | | | | | | | | | | | | | | | | | | | | | |  |  |  |  |  |
| **Comparison** | | | | | | | | |  | | | | | | | | | | | | | | | | | | | | | | | | | | |  |  |  |  |  |
| **Model** | |  | | **Model** | | | | | **ΔR²** | | | | | **F** | | | | | | | | **df1** | | | | | **df2** | | | | | **p** | | | |  |  |  |  |  |
| 1 |  | - |  | 2 | |  | | | 0.18 | |  | | | 246.50 | | | | | |  | | 1 | | |  | | 1006 | | | |  | < .001 | | |  |  |  |  |  |  |
| 2 |  | - |  | 3 | |  | | | 0.00 | |  | | | 0.49 | | | | | |  | | 3 | | |  | | 1003 | | | |  | 0.689 | | |  |  |  |  |  |  |
|  | | | | | | | | | | | | | | | | | | | | | | | | | | | | | | | | | | | |  |  |  |  |  |
| **Step 1** | | | | | | | | | | | | | | | | | | | | | | | | | | | | | | | | | | | | | | | | |
|  | | | | | | | | | | | | | | | | | | | | | | | | | | | | | | | | | | | | | **95% Confidence Interval** | | | |
| **Predictor** | | | | | | | | **Estimate** | | | | | **SE** | | | | | **t** | | | | | | **p** | | | | | **Stand. Estimate** | | | | | | | | **Lower** | | **Upper** | |
| Intercept ᵃ | | | | |  | | | 4.65 | | | |  | 0.07 | | | |  | 70.74 | | | | |  | < .001 | | | |  |  | | | |  | | | |  |  |  |  |
| gender: | | | | |  | | |  | | | |  |  | | | |  |  | | | | |  |  | | | |  |  | | | |  | | | |  |  |  |  |
| Female – Male | | | | |  | | | 0.32 | | | |  | 0.06 | | | |  | 5.25 | | | | |  | < .001 | | | |  | 0.31 | | | |  | | | | 0.20 |  | 0.43 |  |
| age: | | | | |  | | |  | | | |  |  | | | |  |  | | | | |  |  | | | |  |  | | | |  | | | |  |  |  |  |
| 35-54 yrs – < 35 yrs | | | | |  | | | 0.31 | | | |  | 0.07 | | | |  | 4.13 | | | | |  | < .001 | | | |  | 0.30 | | | |  | | | | 0.16 |  | 0.45 |  |
| > 54 yrs – < 35 yrs | | | | |  | | | 0.72 | | | |  | 0.08 | | | |  | 8.93 | | | | |  | < .001 | | | |  | 0.71 | | | |  | | | | 0.56 |  | 0.87 |  |
| ᵃ Represents reference level | | | | | | | | | | | | | | | | | | | | | | | | | | | | | | | | | | | | | | | | |
|  | | | | | | | | | | | | | | | | | | | | | | | | | | | | | | | | | | | | | | | | |

| **Step 2** | | | | | | | | | | | | | | | |
| --- | --- | --- | --- | --- | --- | --- | --- | --- | --- | --- | --- | --- | --- | --- | --- |
|  | | | | | | | | | | | | **95% Confidence Interval** | | | |
| **Predictor** | | **Estimate** | | **SE** | | **t** | | **p** | | **Stand. Estimate** | | **Lower** | | **Upper** | |
| Intercept ᵃ |  | 2.39 |  | 0.16 |  | 15.35 |  | < .001 |  |  |  |  |  |  |  |
| gender: |  |  |  |  |  |  |  |  |  |  |  |  |  |  |  |
| Female – Male |  | 0.22 |  | 0.05 |  | 4.00 |  | < .001 |  | 0.22 |  | 0.11 |  | 0.32 |  |
| age: |  |  |  |  |  |  |  |  |  |  |  |  |  |  |  |
| 35-54 yrs – < 35 yrs |  | 0.25 |  | 0.07 |  | 3.73 |  | < .001 |  | 0.25 |  | 0.12 |  | 0.38 |  |
| > 54 yrs – < 35 yrs |  | 0.62 |  | 0.07 |  | 8.51 |  | < .001 |  | 0.61 |  | 0.47 |  | 0.75 |  |
| hc_score |  | 0.45 |  | 0.03 |  | 15.70 |  | < .001 |  | 0.43 |  | 0.37 |  | 0.48 |  |
| ᵃ Represents reference level | | | | | | | | | | | | | | | |
|  | | | | | | | | | | | | | | | |

The final model including the 3^rd^ regression step is reported in the main text.

 [b] beh_ener

| Model Fit Measures | | | | | | | | | | | |
| --- | --- | --- | --- | --- | --- | --- | --- | --- | --- | --- | --- |
|  | | | | **Overall Model Test** | | | | | | | |
| **Model** | | **Adjusted R²** | | **F** | | **df1** | | **df2** | | **p** | |
| 1 |  | 0.07 |  | 26.09 |  | 3 |  | 1007 |  | < .001 |  |
| 2 |  | 0.20 |  | 63.65 |  | 4 |  | 1006 |  | < .001 |  |
| 3 |  | 0.20 |  | 37.04 |  | 7 |  | 1003 |  | < .001 |  |
|  | | | | | | | | | | | |

| Model Comparisons | | | | | | | | | | | | | | | |
| --- | --- | --- | --- | --- | --- | --- | --- | --- | --- | --- | --- | --- | --- | --- | --- |
| **Comparison** | | | | | |  | | | | | | | | | |
| **Model** | |  | | **Model** | | **ΔR²** | | **F** | | **df1** | | **df2** | | **p** | |
| 1 |  | - |  | 2 |  | 0.13 |  | 163.67 |  | 1 |  | 1006 |  | < .001 |  |
| 2 |  | - |  | 3 |  | 0.00 |  | 1.45 |  | 3 |  | 1003 |  | 0.227 |  |
|  | | | | | | | | | | | | | | | |

| **Step 1** | | | | | | | | | | | | | | | |
| --- | --- | --- | --- | --- | --- | --- | --- | --- | --- | --- | --- | --- | --- | --- | --- |
|  | | | | | | | | | | | | **95% Confidence Interval** | | | |
| **Predictor** | | **Estimate** | | **SE** | | **t** | | **p** | | **Stand. Estimate** | | **Lower** | | **Upper** | |
| Intercept ᵃ |  | 5.36 |  | 0.07 |  | 76.17 |  | < .001 |  |  |  |  |  |  |  |
| gender: |  |  |  |  |  |  |  |  |  |  |  |  |  |  |  |
| Female – Male |  | 0.20 |  | 0.06 |  | 3.09 |  | 0.002 |  | 0.19 |  | 0.07 |  | 0.31 |  |
| age: |  |  |  |  |  |  |  |  |  |  |  |  |  |  |  |
| 35-54 yrs – < 35 yrs |  | 0.33 |  | 0.08 |  | 4.13 |  | < .001 |  | 0.31 |  | 0.16 |  | 0.46 |  |
| > 54 yrs – < 35 yrs |  | 0.70 |  | 0.09 |  | 8.12 |  | < .001 |  | 0.66 |  | 0.50 |  | 0.82 |  |
| ᵃ Represents reference level | | | | | | | | | | | | | | | |
|  | | | | | | | | | | | | | | | |

| **Step 2** | | | | | | | | | | | | | | | |
| --- | --- | --- | --- | --- | --- | --- | --- | --- | --- | --- | --- | --- | --- | --- | --- |
|  | | | | | | | | | | | | **95% Confidence Interval** | | | |
| **Predictor** | | **Estimate** | | **SE** | | **t** | | **p** | | **Stand. Estimate** | | **Lower** | | **Upper** | |
| Intercept ᵃ |  | 3.32 |  | 0.17 |  | 19.26 |  | < .001 |  |  |  |  |  |  |  |
| gender: |  |  |  |  |  |  |  |  |  |  |  |  |  |  |  |
| Female – Male |  | 0.11 |  | 0.06 |  | 1.83 |  | 0.068 |  | 0.10 |  | -0.01 |  | 0.22 |  |
| age: |  |  |  |  |  |  |  |  |  |  |  |  |  |  |  |
| 35-54 yrs – < 35 yrs |  | 0.28 |  | 0.07 |  | 3.74 |  | < .001 |  | 0.26 |  | 0.12 |  | 0.40 |  |
| > 54 yrs – < 35 yrs |  | 0.61 |  | 0.08 |  | 7.56 |  | < .001 |  | 0.57 |  | 0.42 |  | 0.72 |  |
| hc_score |  | 0.41 |  | 0.03 |  | 12.79 |  | < .001 |  | 0.36 |  | 0.31 |  | 0.42 |  |
| ᵃ Represents reference level | | | | | | | | | | | | | | | |
|  | | | | | | | | | | | | | | | |

 The final model including the 3^rd^ regression step is reported in the main text.

[c] beh_sust

| Model Fit Measures | | | | | | | | | | | |
| --- | --- | --- | --- | --- | --- | --- | --- | --- | --- | --- | --- |
|  | | | | **Overall Model Test** | | | | | | | |
| **Model** | | **Adjusted R²** | | **F** | | **df1** | | **df2** | | **p** | |
| 1 |  | 0.02 |  | 9.16 |  | 3 |  | 1007 |  | < .001 |  |
| 2 |  | 0.12 |  | 34.25 |  | 4 |  | 1006 |  | < .001 |  |
| 3 |  | 0.11 |  | 19.73 |  | 7 |  | 1003 |  | < .001 |  |
|  | | | | | | | | | | | |

| Model Comparisons | | | | | | | | | | | | | | | |
| --- | --- | --- | --- | --- | --- | --- | --- | --- | --- | --- | --- | --- | --- | --- | --- |
| **Comparison** | | | | | |  | | | | | | | | | |
| **Model** | |  | | **Model** | | **ΔR²** | | **F** | | **df1** | | **df2** | | **p** | |
| 1 |  | - |  | 2 |  | 0.09 |  | 106.63 |  | 1 |  | 1006 |  | < .001 |  |
| 2 |  | - |  | 3 |  | 0.00 |  | 0.45 |  | 3 |  | 1003 |  | 0.720 |  |
|  | | | | | | | | | | | | | | | |

| **Step 1** | | | | | | | | | | | | | | | |
| --- | --- | --- | --- | --- | --- | --- | --- | --- | --- | --- | --- | --- | --- | --- | --- |
|  | | | | | | | | | | | | **95% Confidence Interval** | | | |
| **Predictor** | | **Estimate** | | **SE** | | **t** | | **p** | | **Stand. Estimate** | | **Lower** | | **Upper** | |
| Intercept ᵃ |  | 4.31 |  | 0.07 |  | 63.78 |  | < .001 |  |  |  |  |  |  |  |
| gender: |  |  |  |  |  |  |  |  |  |  |  |  |  |  |  |
| Female – Male |  | 0.12 |  | 0.06 |  | 1.96 |  | 0.050 |  | 0.12 |  | 0.00 |  | 0.24 |  |
| age: |  |  |  |  |  |  |  |  |  |  |  |  |  |  |  |
| 35-54 yrs – < 35 yrs |  | 0.18 |  | 0.08 |  | 2.38 |  | 0.017 |  | 0.18 |  | 0.03 |  | 0.33 |  |
| > 54 yrs – < 35 yrs |  | 0.39 |  | 0.08 |  | 4.75 |  | < .001 |  | 0.39 |  | 0.23 |  | 0.56 |  |
| ᵃ Represents reference level | | | | | | | | | | | | | | | |
|  | | | | | | | | | | | | | | | |

| **Step 2** | | | | | | | | | | | | | | | |
| --- | --- | --- | --- | --- | --- | --- | --- | --- | --- | --- | --- | --- | --- | --- | --- |
|  | | | | | | | | | | | | **95% Confidence Interval** | | | |
| **Predictor** | | **Estimate** | | **SE** | | **t** | | **p** | | **Stand. Estimate** | | **Lower** | | **Upper** | |
| Intercept ᵃ |  | 2.69 |  | 0.17 |  | 15.84 |  | < .001 |  |  |  |  |  |  |  |
| gender: |  |  |  |  |  |  |  |  |  |  |  |  |  |  |  |
| Female – Male |  | 0.05 |  | 0.06 |  | 0.85 |  | 0.394 |  | 0.05 |  | -0.07 |  | 0.17 |  |
| age: |  |  |  |  |  |  |  |  |  |  |  |  |  |  |  |
| 35-54 yrs – < 35 yrs |  | 0.14 |  | 0.07 |  | 1.93 |  | 0.054 |  | 0.14 |  | -0.00 |  | 0.28 |  |
| > 54 yrs – < 35 yrs |  | 0.32 |  | 0.08 |  | 4.04 |  | < .001 |  | 0.32 |  | 0.16 |  | 0.48 |  |
| hc_score |  | 0.33 |  | 0.03 |  | 10.33 |  | < .001 |  | 0.31 |  | 0.25 |  | 0.37 |  |
| ᵃ Represents reference level | | | | | | | | | | | | | | | |
|  | | | | | | | | | | | | | | | |

  The final model including the 3^rd^ regression step is reported in the main text.

[d] beh_recy

| Model Fit Measures | | | | | | | | | | | |
| --- | --- | --- | --- | --- | --- | --- | --- | --- | --- | --- | --- |
|  | | | | **Overall Model Test** | | | | | | | |
| **Model** | | **Adjusted R²** | | **F** | | **df1** | | **df2** | | **p** | |
| 1 |  | 0.06 |  | 21.41 |  | 3 |  | 1007 |  | < .001 |  |
| 2 |  | 0.20 |  | 65.11 |  | 4 |  | 1006 |  | < .001 |  |
| 3 |  | 0.20 |  | 38.04 |  | 7 |  | 1003 |  | < .001 |  |
|  | | | | | | | | | | | |

| Model Comparisons | | | | | | | | | | | | | | | |
| --- | --- | --- | --- | --- | --- | --- | --- | --- | --- | --- | --- | --- | --- | --- | --- |
| **Comparison** | | | | | |  | | | | | | | | | |
| **Model** | |  | | **Model** | | **ΔR²** | | **F** | | **df1** | | **df2** | | **p** | |
| 1 |  | - |  | 2 |  | 0.15 |  | 184.52 |  | 1 |  | 1006 |  | < .001 |  |
| 2 |  | - |  | 3 |  | 0.00 |  | 1.75 |  | 3 |  | 1003 |  | 0.155 |  |
|  | | | | | | | | | | | | | | | |

| **Step 1** | | | | | | | | | | | | | | | |
| --- | --- | --- | --- | --- | --- | --- | --- | --- | --- | --- | --- | --- | --- | --- | --- |
|  | | | | | | | | | | | | **95% Confidence Interval** | | | |
| **Predictor** | | **Estimate** | | **SE** | | **t** | | **p** | | **Stand. Estimate** | | **Lower** | | **Upper** | |
| Intercept ᵃ |  | 5.23 |  | 0.07 |  | 72.64 |  | < .001 |  |  |  |  |  |  |  |
| gender: |  |  |  |  |  |  |  |  |  |  |  |  |  |  |  |
| Female – Male |  | 0.13 |  | 0.07 |  | 1.89 |  | 0.059 |  | 0.12 |  | -0.00 |  | 0.24 |  |
| age: |  |  |  |  |  |  |  |  |  |  |  |  |  |  |  |
| 35-54 yrs – < 35 yrs |  | 0.24 |  | 0.08 |  | 2.95 |  | 0.003 |  | 0.22 |  | 0.07 |  | 0.37 |  |
| > 54 yrs – < 35 yrs |  | 0.66 |  | 0.09 |  | 7.51 |  | < .001 |  | 0.61 |  | 0.45 |  | 0.77 |  |
| ᵃ Represents reference level | | | | | | | | | | | | | | | |
|  | | | | | | | | | | | | | | | |

| **Step 2** | | | | | | | | | | | | | | | |
| --- | --- | --- | --- | --- | --- | --- | --- | --- | --- | --- | --- | --- | --- | --- | --- |
|  | | | | | | | | | | | | **95% Confidence Interval** | | | |
| **Predictor** | | **Estimate** | | **SE** | | **t** | | **p** | | **Stand. Estimate** | | **Lower** | | **Upper** | |
| Intercept ᵃ |  | 3.03 |  | 0.17 |  | 17.35 |  | < .001 |  |  |  |  |  |  |  |
| gender: |  |  |  |  |  |  |  |  |  |  |  |  |  |  |  |
| Female – Male |  | 0.03 |  | 0.06 |  | 0.47 |  | 0.641 |  | 0.03 |  | -0.08 |  | 0.14 |  |
| age: |  |  |  |  |  |  |  |  |  |  |  |  |  |  |  |
| 35-54 yrs – < 35 yrs |  | 0.19 |  | 0.08 |  | 2.46 |  | 0.014 |  | 0.17 |  | 0.03 |  | 0.31 |  |
| > 54 yrs – < 35 yrs |  | 0.56 |  | 0.08 |  | 6.91 |  | < .001 |  | 0.52 |  | 0.37 |  | 0.67 |  |
| hc_score |  | 0.44 |  | 0.03 |  | 13.58 |  | < .001 |  | 0.39 |  | 0.33 |  | 0.44 |  |
| ᵃ Represents reference level | | | | | | | | | | | | | | | |
|  | | | | | | | | | | | | | | | |

 The final model including the 3^rd^ regression step is reported in the main text.

[e] beh_mobi

| Model Fit Measures | | | | | | | | | | | |
| --- | --- | --- | --- | --- | --- | --- | --- | --- | --- | --- | --- |
|  | | | | **Overall Model Test** | | | | | | | |
| **Model** | | **Adjusted R²** | | **F** | | **df1** | | **df2** | | **p** | |
| 1 |  | 0.00 |  | 0.04 |  | 3 |  | 1007 |  | 0.989 |  |
| 2 |  | 0.03 |  | 8.83 |  | 4 |  | 1006 |  | < .001 |  |
| 3 |  | 0.03 |  | 5.22 |  | 7 |  | 1003 |  | < .001 |  |
|  | | | | | | | | | | | |

| Model Comparisons | | | | | | | | | | | | | | | |
| --- | --- | --- | --- | --- | --- | --- | --- | --- | --- | --- | --- | --- | --- | --- | --- |
| **Comparison** | | | | | |  | | | | | | | | | |
| **Model** | |  | | **Model** | | **ΔR²** | | **F** | | **df1** | | **df2** | | **p** | |
| 1 |  | - |  | 2 |  | 0.03 |  | 35.20 |  | 1 |  | 1006 |  | < .001 |  |
| 2 |  | - |  | 3 |  | 0.00 |  | 0.43 |  | 3 |  | 1003 |  | 0.729 |  |
|  | | | | | | | | | | | | | | | |

| **Step 1** | | | | | | | | | | | | | | | |
| --- | --- | --- | --- | --- | --- | --- | --- | --- | --- | --- | --- | --- | --- | --- | --- |
|  | | | | | | | | | | | | **95% Confidence Interval** | | | |
| **Predictor** | | **Estimate** | | **SE** | | **t** | | **p** | | **Stand. Estimate** | | **Lower** | | **Upper** | |
| Intercept ᵃ |  | 3.82 |  | 0.08 |  | 45.03 |  | < .001 |  |  |  |  |  |  |  |
| gender: |  |  |  |  |  |  |  |  |  |  |  |  |  |  |  |
| Female – Male |  | -0.01 |  | 0.08 |  | -0.16 |  | 0.874 |  | -0.01 |  | -0.13 |  | 0.11 |  |
| age: |  |  |  |  |  |  |  |  |  |  |  |  |  |  |  |
| 35-54 yrs – < 35 yrs |  | 0.03 |  | 0.10 |  | 0.31 |  | 0.753 |  | 0.02 |  | -0.13 |  | 0.18 |  |
| > 54 yrs – < 35 yrs |  | 0.02 |  | 0.10 |  | 0.17 |  | 0.867 |  | 0.01 |  | -0.15 |  | 0.18 |  |
| ᵃ Represents reference level | | | | | | | | | | | | | | | |
|  | | | | | | | | | | | | | | | |

| **Step 2** | | | | | | | | | | | | | | | |
| --- | --- | --- | --- | --- | --- | --- | --- | --- | --- | --- | --- | --- | --- | --- | --- |
|  | | | | | | | | | | | | **95% Confidence Interval** | | | |
| **Predictor** | | **Estimate** | | **SE** | | **t** | | **p** | | **Stand. Estimate** | | **Lower** | | **Upper** | |
| Intercept ᵃ |  | 2.61 |  | 0.22 |  | 11.85 |  | < .001 |  |  |  |  |  |  |  |
| gender: |  |  |  |  |  |  |  |  |  |  |  |  |  |  |  |
| Female – Male |  | -0.07 |  | 0.08 |  | -0.85 |  | 0.396 |  | -0.05 |  | -0.18 |  | 0.07 |  |
| age: |  |  |  |  |  |  |  |  |  |  |  |  |  |  |  |
| 35-54 yrs – < 35 yrs |  | -0.00 |  | 0.09 |  | -0.01 |  | 0.994 |  | -0.00 |  | -0.15 |  | 0.15 |  |
| > 54 yrs – < 35 yrs |  | -0.04 |  | 0.10 |  | -0.36 |  | 0.716 |  | -0.03 |  | -0.19 |  | 0.13 |  |
| hc_score |  | 0.24 |  | 0.04 |  | 5.93 |  | < .001 |  | 0.19 |  | 0.12 |  | 0.25 |  |
| ᵃ Represents reference level | | | | | | | | | | | | | | | |
|  | | | | | | | | | | | | | | | |

 The final model including the 3^rd^ regression step is reported in the main text.
